# Effect of cochlear implant surgery on vestibular function: meta-analysis study

**DOI:** 10.1186/s40463-017-0224-0

**Published:** 2017-06-08

**Authors:** Iman Ibrahim, Sabrina Daniela da Silva, Bernard Segal, Anthony Zeitouni

**Affiliations:** 1 Department of Otolaryngology-Head and Neck Surgery, Royal Victoria Hospital, McGill University, Montréal, Canada; 2 Department of Otolaryngology-Head and Neck Surgery, Jewish General Hospital, McGill University, Montréal, Canada

**Keywords:** Cochlear Implant, Vestibular function, Postural stability, Vestibular disorders

## Abstract

**Importance:**

Vestibular disorders have been reported following cochlear implant (CI) surgery, but the literature shows a wide discrepancy in the reported clinical impact. The aim of this meta-analysis is to quantify the effect of CI before and after surgery on the outcomes of vestibular tests, postural stability, and subjective perception of dizziness.

**Objective:**

To evaluate the effects of CI surgery on vestibular function in adult patients (≥18 years) with sensorineural hearing loss who underwent unilateral or bilateral implantation.

**Data sources:**

MEDLINE, PubMed, Web of Science and Cochrane Library from January 1, 1995, through July 12, 2016.

**Study selection:**

Published studies of adult patients who received unilateral or bilateral CIs and whose vestibular function or postural stability was assessed before and after surgery.

**Data extraction:**

From each study, test results before and after surgery were compared, for the following five tests: clinical head impulse test (HIT); bi-thermal caloric irrigation of the horizontal semicircular canal; vestibular evoked myogenic potential (VEMP); dizziness handicap inventory (DHI); and computerized dynamic posturography (CDP).

**Results:**

Twenty-seven studies met all inclusion criteria. Most studies performed either bi-thermal caloric irrigation and/or VEMP, with fewer studies investigating changes in HIT, posturography or DHI. CI surgery significantly affected the results of caloric and VEMP testing. However, HIT results, posturography, and DHI, scores were not significantly affected after CI surgery.

**Conclusions and relevance:**

CI surgery has a significant negative effect on the results of caloric as well as VEMP tests. No significant effect of CI surgery was detected in HIT, posturography, or DHI scores. Overall, the clinical effect of CI surgery on the vestibular function was found to be insignificant. Nonetheless, the potential effects of surgery on the vestibular system should be discussed with CI candidates before surgery.

## Background

Hearing loss is the most common sensory deficit of all. More than 5% of the world’s population suffer from disabling hearing loss, affecting about one-third of people above 65 years of age [1]. In cases where hearing aids are no longer useful or sufficient, cochlear implant (CI) surgery is the standard procedure for the treatment of hearing loss. CI attempts to replace the function of hair cells that are no longer able to stimulate primary auditory neurons in response to sound. While the effects of CI surgery on residual cochlear function is well studied, less attention has been given to its effects on vestibular function. Such effects occur because CI surgery frequently affects the vestibular apparatus, which is in close anatomical proximity to the auditory system.

Different mechanisms that could lead to vestibular dysfunction during or after CI surgery have been postulated: 1) direct trauma caused by electrode insertion, 2) acute serous labyrinthitis due to cochleostomy, 3) foreign body reaction with labyrinthitis, 4) endolymphatic hydrops, and 5) electrical stimulation from the implant itself [2].

The occurrence of vestibular dysfunction following CI surgery has a very wide range as assessed by bi-thermal caloric testing and vestibular evoked myogenic potential (VEMP) testing [2–6]. However, not all CI recipients suffer from postoperative dizziness [2–5], and CI recipients reported different forms of dizziness after surgery. Postoperative dizziness had different characteristics, onset, and duration [6].

Given the increasing use of bilateral implantation, it would be important to be able to quantify the effects of CI surgery on the vestibular system. This information would be of great benefit both to the CI team and patients. The aim of the current study was to evaluate the effects of CI surgery on vestibular function and postural stability in adult patients having sensorineural hearing loss (SNHL) who underwent unilateral or bilateral implantation. The purpose of the current meta-analysis study was to demonstrate a quantifiable effect of CI surgery on several tests for balance and vestibular function.

## Methods

PRISMA (Preferred Reporting Items for Systematic Reviews and Meta-analysis) statement was used as our methodology for this systematic review [7].

### Study eligibility criteria

The criteria used in the selection were: (1) studies including adult patients (≥18 years old), (2) studies reporting both pre- and postoperative test results, and (3) studies that reported numbers of normal and abnormal patients for the following tests: clinical head impulse test (HIT), caloric, and vestibular evoked myogenic potential (VEMP) testing were included. Studies that reported raw or average data and standard deviations for posturography (Sensory Organization Test (SOT) conditions 5 and 6) or for dizziness handicap inventory (DHI) pre- and postoperatively were also included. Studies involving young patients (<18 years) were excluded.

All studies had CI surgery performed by the same surgical unit, so it was assumed that the techniques between patients were standardized.

### Data sources

A thorough search of MEDLINE, PubMed, EMBASE, Web of Science and Cochrane Review was conducted, using the keywords “cochlear implant and vestibular” or “cochlear implant and caloric” or “cochlear implant and VEMP” or “cochlear implant and balance” or “cochlear implant and posturography” or “cochlear implant and dizziness” or “cochlear implant and Dizziness Handicap Inventory”. This meta-analysis included the date range from January 1^st^, 1995 to July 12^th^, 2016.

### Data extraction

A total of 2006 potential journal articles was identified using the keywords mentioned above. Only articles in English and French were included. Individual studies’ abstracts were screened to select the studies that met the criteria for this meta-analysis. Full texts of selected articles were retrieved and then rescreened for possible inclusion in the current meta-analysis by two different observers independently.

### Data presentation

Different tests exist to evaluate different aspects of the state of the vestibular apparatus. The HIT is one test that assesses vestibulo-ocular function. Other tests objectively evaluate parameters associated with different parts of the vestibular apparatus; however, they do not measure the function of the vestibular system. Such tests include the caloric and VEMP tests.

Posturography is a set of tests that assess the integrative vestibular performance associated with maintenance of posture, where the vestibular function integrates with other sensory inputs (such as vision & proprioception, in order to maintain posture). When applying the SOT test, posturography assesses the state of compensation, because all the movements are sway-referenced, with no induced movements. The DHI is a subjective test for assessment of the perceived function of the vestibular balance condition.

### Data synthesis

Four separate meta-analyses were conducted - one for each test. For HIT, caloric, and VEMP testing, the outcome measure was obtained from the ratio of subjects with normal test results before and after surgery; the effect size was measured using the log relative risk (RR) because outcomes are reported in a dichotomous manner (i.e. either normal or hypo/areflexia). For Posturography and DHI, the outcome measure was the mean difference in scores; the effect size was measured using the mean difference (MD) in scores before and after surgery. The random effects model was used, because of the expected variability in the tests’ conditions and results interpretation in the different test centers, and also because all the heterogeneity analyses were significant. Due to the low number of studies available, a meta-analysis was not performed for the posturography data. To calculate the mean difference in scores, the means and standard deviations for scores were extracted, as well as the number of subjects before and after surgery. All data analyses were performed using R-version-3.1.2. Statistical significance was defined as *P* < 0.05.

## Results

**Fig. 1:**
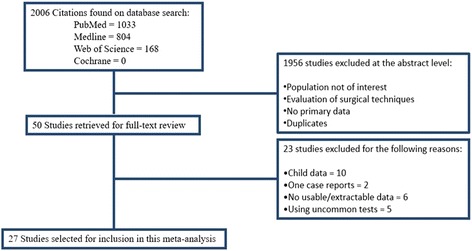
Flow diagram of search and study selection process

**Fig. 2:**
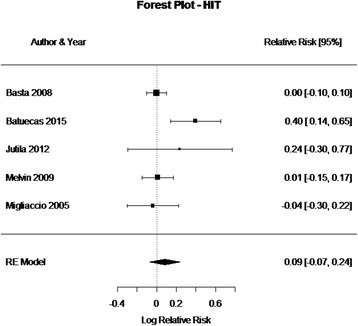
Forest plot (showing relative effect sizes) for the HIT test

**Fig. 3:**
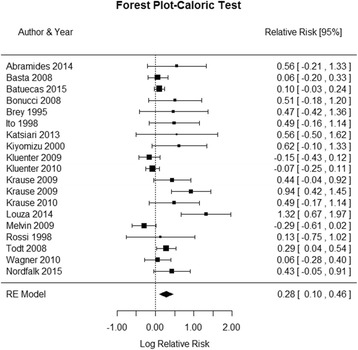
Forest plot (showing relative effect sizes) for the caloric test

**Fig. 4:**
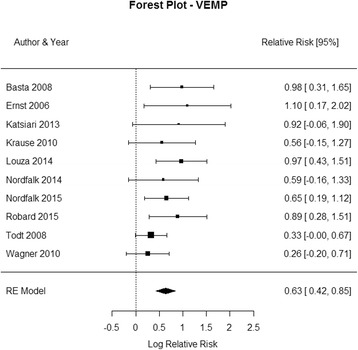
Forest plot (showing relative effect sizes) for the VEMP test

**Fig. 5:**
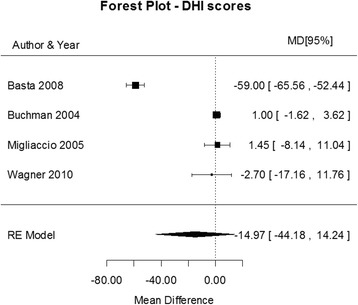
Forest plot (showing relative effect sizes) for the DHI test

Of the 2006 studies, 1956 articles were excluded at the abstract level because they were either duplicates or because the eligibility criteria did not apply (Fig. 1). Next, the full-text of 50 publications were recovered, and then 23 of these publications were excluded because it was not possible to extract useful data from them. Those reports either did not report numbers of subjects having preoperative normal vestibular function and/or numbers of subjects having postoperative normal vestibular functions, or they applied different forms of tests not evaluated in this study. The remaining 27 reports were included in the meta-analysis (Fig. 1) and the results were described separately (Table 1, Figs. 2, 3, 4 and 5).

**Table 1:** Summary of results of all studies included in the meta-analyses

Source (publication)	Study design	Follow-up (days)	Number of patients	Mean age (range)	HIT + RE	Caloric + RE	VEMP + RE	DHI+ RE	CDP + RE
Abramides 2015 [18], Sao Paolo, Brazil	Prospective study	120	24	42 (12–65)		Yes *P* = 0.414			
Basta 2008 [12] Berlin, Germany	Prospective study	42	18	(10–75)	Yes ND (NS)	Yes ND (NS)	Yes *P* < 0.05	Yes ND (NS)	
Bateucas 2015 [8] Salamanca, Spain	Prospective descriptive	2	30	54 ± 10	Yes	Yes			
Bonucci 2008 [15] Sao Paolo, Brazil	NI*	NI*	38	30.65 ± 32 4–62		Yes ND			
Brey 1995 [14] Mayo clinic,Rochester, Minnesota	NI*	45 to 1770	52	3-87		Yes P = 0.01			Yes ND
Buchman 2004 [3] University of North Carolina, USA	Prospectivestudy	30	67	2-87				Yes ND	Yes ND
Coordes 2012 [13] Berlin, Germany	Prospective study	NI*	17	60 (20–73)			Yes ND		
Ernst 2006 [30] Berlin, Germany	Prospective study	365	18	18-62			Yes ND (NS)		
Ito 1998 [31] Otsu, Japan	NI*	30	55	>18		Yes ND			
Jutila 2012 [32] Helsinki, Finland	Prospective study	60	44	55 (30–76)	Yes P > 0.05				
Katsiari 2013 [2] Piraeus, Greece	Prospective study	30	20	47.6 ± 20.2 10–77		Yes *P* = 0.01	Yes *P* = 0.002		
Kiyomizu 2000 [33] Miyazaki, Japan	NI*	NI*	23	36-75		Yes ND			
Kluenter 2009 [6] Fena, Germany	Prospective study	42 31–368)	52	47(11–74)		Yes ND			
Kluenter 2010 [25] Fena, Germany	Prospective study	44 (31–363)	24	51 (20–75)		Yes ND			
Krause 2009a [22] Munich, Germany	Prospective study	28 - 42	59	54 (15–83)		Yes *P* < 0.001			
Krause 2009b [23] Munich, Germany	Prospectivestudy	28	47	54 (16–83)		Yes *P* < 0.01			
Krause 2010 [24] Munich, Germany	Prospectivestudy	60	32	55 (15–83)		Yes *P* < 0.001	Yes *P* < 0.047		
Louza 2015 [34] Munich, Germany	Retrospective observational study	28 - 42	41	>14 56 ± 19		Yes ND	Yes ND		
Melvin 2009 [5] Johns Hopkins, Maryland, USA	Prospective cohort	28 - 42	16	46 (23–69)	Yes ND	Yes ND	Yes ND		
Migliaccio 2005 [10] Johns Hopkins, Maryland, USA	Prospective study	28 - 42	16	46 (27–64)	Yes *P* > 0.05				
Nordfalk 2014 [21] Oslo, Norway	Prospective pilot	28 - 42	12	32-61			Yes ND		
Nordfalk 2015 [19] Oslo, Norway	Prospective	42-56	39	57.5 ± 17.2 (18–83)		Yes ND	Yes ND		
Robard 2015 [11] Caen, France	Prospective study	150	34	49 ± 25 (1–86)			Yes *P* = 0.0015		
Rossi 1998 [35] Turin, Italy	Case series	180	32	12-74		Yes ND			
Todt 2008 [36] Berlin, Germany	Retrospective cohort	42 - 56	62	17-84		Yes ND	Yes ND		
Vankatova 2014 [9] Geneve, Switzerland	Retrospective study	NI*	50	15-72	Yes ND	Yes ND			
Wagner 2010 [17] Berlin, Germany	Retrospective cohort	42 - 56	20	41.5 (11–58)		Yes ND	Yes ND		

HIT* head impulse test, VEMP* vestibular evoked myogenic potential, DHI* dizziness handicap inventory, CDP* computerized dynamic posturography, RE* reported effect, NI* not identified. ND* not detected, NS* non-significant, S* significant. RE* reported effect

### HIT results

The number of subjects with normal and abnormal testing results before and after CI surgery who were included in the meta-analysis of the HIT test is shown in Table 2 in Appendix. The statistical analysis revealed a non-significant effect of CI surgery on the HIT test results (RR = 0.0951, 95% CI = −0.0220, 0.2122, *P* = 0.11). There was substantial variability in the results observed in these studies (I^2 =^ 57.98%, QDF = 5) = 11.2612, *P* = 0.046). The forest plot indicating the relative strength of each study included in the meta-analysis is illustrated by Fig. 2. Two studies (Batuecas et al., [8] and Vankatova et al. [9]) had a relatively larger number of abnormal postoperative HIT results. However, patients in Batuecas et al. [8] were re-tested after a relatively short postoperative period (2 days). For Vankatova et al. [9], communication with the authors revealed that they had false positive results. Consequently, it was decided to exclude this study from the meta-analysis.

Five out of the six studies that performed HIT, conducted a quantitative HIT, whether a video HIT [8, 9], a search coil HIT [5, 10], or a motorized HIT (mHIT) [11]. Only Basta et al., [12] used a bedside HIT.

### Caloric test results

The number of subjects with normal and abnormal testing results before and after CI surgery included in the meta-analysis of the caloric test is shown in Table 3 in Appendix. The statistical analysis revealed a significant effect of CI surgery on the caloric test results (RR = 0.2826, 95% CI = 0.1032, 0.4621, *P* = 0.0039). There was a considerable heterogeneity observed in the studies (I^2 =^ 74.90%, Q (DF = 18) = 50.8956, *P* < 0.0001). The forest plot indicating the relative strength of each study included in the meta-analysis is illustrated by Fig. 3. Despite the variability among the reports, the results revealed a tendency for loss of peripheral vestibular function following CI surgery in the majority of the 19 studies involved in this analysis. Several factors could account for the variability among the studies, such as the age range, the test settings and timing of the postoperative retest.

### VEMP test results

The studies included in the meta-analysis of VEMP test are shown in Table 4 in Appendix. All included studies used cVEMP. The statistical analysis revealed a significant detrimental effect of CI surgery on VEMP test results (RR = 0.5099, 95% CI = 0.2941, 0.7256, *P* < 0.0001). There was a substantial heterogeneity in the studies (I^2 =^ 51.68%, Q (DF = 11) = 20.7693, *P* = 0.0293). The forest plot indicating the relative strength of each study included in the meta-analysis is illustrated by Fig. 4. Two studies (Coordes et al. [13], and Melvin et al. [5]) had a relatively higher number of patients who retained normal VEMP test results postoperatively. This could be due to the use of bone-conduction VEMP, which is more sensitive compared to air-conduction VEMP [13].

### Posturography results

The results from the studies that investigated posturography, particularly the conditions 5 and 6 are shown in Table 5 in Appendix. Meta-analysis could not be conducted because only two studies were retrieved [3, 14]. Brey et al. [14]. found a non-significant difference between pre- and post- implantation, where the difference in conditions 5 and 6 scores was very subtle: These results did not differ much from the results reported by Buchman et al. [3]. Overall, postural stability performance did not seem to be affected by the CI surgery.

### DHI results

Results from the studies that were included in the meta-analysis of the DHI test are shown in Table 6 in Appendix. The statistical analysis revealed a non-significant effect of CI surgery on the DHI scores (MD = −14.9718, 95% CI = −44.1804, 14.23, *P* = 0.3151). There was a considerable heterogeneity in the studies (I^2 =^ 98.65%, Q (df = 3) = 280.0102, *P*.0001). The forest plot showing the relative strength of each study included in the meta-analysis is illustrated by Fig. 5. Basta et al., [12] reported an unusually high postoperative mean score. However, these authors analyzed only five patients with a significant increase in their DHI scores after the surgery. All of them were significantly older (68.8 ± 6.5 years), as compared to the other studies (mean 46.7 ± 18.2 years). Results from DHI scores agree with posturography results, where in most studies, even those reporting increased DHI scores did not result in a change that required further investigation and/or intervention.

## Discussion

Vestibular disorders have been reported following CI surgery. This systematic review and meta-analysis showed great variability in the tests’ results. This variability might be due to the different testing measures employed. Both HIT and caloric tests are strongly affected by the lateral semicircular canal function. VEMP testing is strongly influenced by the saccular function. Posturography testing is closely related to the compensatory mechanisms of postural performance. DHI assessments characterize a patient’s subjective impression about their balance perception. Thus it appears that CI may affect some aspects of vestibular function [5]. The variability may also be partly explained by the differences in the criteria and/or test techniques such as the cut-off to determine the normal versus abnormal test results [2]. However, not all studies reported their criteria.

Two studies [8, 9] had a relatively larger number of abnormal postoperative HIT results. Maybe this can be explained by the short postoperative re-test period (2 days) [8]. Unfortunally, was not possible to pool and analyze studies based on follow-up periods because several papers were not specific, either they did not specify the period [15], or provided a very wide range for it [14].

For VEMP results, two studies [5, 13] showed better postoperative results. This could be due to the use of bone-conduction VEMP, which is more sensitive compared to air-conduction VEMP [13], and hence were not included in the meta-analysis. For DHI results, Basta et al., [12] reported an unusually high postoperative mean score. However, these authors analyzed only five patients with a significant increase in their DHI scores after the surgery. All of them were significantly older (68.8 ± 6.5 years), as compared to the other studies (mean 46.7 ± 18.2 years). Results from DHI scores agree with posturography results, where in most studies, even those reporting increased DHI scores did not result in a change that required further investigation and/or intervention.

Another factor that contributes to variability of the results is the fact that CI users are not a homogenous population. They come from different age groups involving newborns to older adults suffering from severe-to-profound SNHL. Thus, age and etiology of SNHL can affect the vestibular function either before, after, or both before and after CI surgery. For example, meningitis often results in disturbed vestibular function due to ossification of the labyrinth (Cushing et al., [16]). From the pooled results in the current meta-analysis, it was found that before surgery, 39.5% had abnormal caloric test results, 31.7% had abnormal VEMP test results, and 11.5% had abnormal HIT results [see Table 1 and Appendix]. Two studies [10, 17] showed a preoperative average DHI scores higher than ten indicating a previous vestibular problem. Few studies reported the number of patients with preoperative caloric or VEMP hyporeflexia who had a deterioration (areflexia) postoperatively [2, 15, 18]. For example, Bonucci et al. [15] found that 10% of the patients who had preoperative hyporeflexia in the caloric test had postoperative areflexia, however, it was not clear whether it was the implanted ear or the contralateral ear. Abramides et al. [18] and Katsiari et al. [2] reported that a deterioration in the non-implanted ear might occur either because the insertion of the electrode in the scala tympani in one ear alters the vestibular input to the brain, and hence modifies the contralateral ear response, or because the reproducibility of the response in these individuals over time is not perfect.

Surgical technique can also affect the outcome. Factors such as electrode insertion site (whether through a cochleostomy, anteroinferior to the round window, or directly through the round window), the electrode length (short or long electrode), the electrode insertion speed, and the electrode insertion depth [19]. The current literature does not provide details about the surgical procedure and only mention the technique used (cochleostomy versus round window approach). The majority of the articles reported the cochleostomy (anteroinferior to the round window) as the standard approach.^1^ Unfortunally, it was not mentioned whether soft surgical techniques were used to minimize trauma to the labyrinth [20].

The data in the current meta-analysis showed no significant increase in DHI in the majority of patients (84.4%), suggesting that CI did not affect balance. Seventy-two percent of the patients retained a normal caloric function after surgery, 60% retained normal HIT results, and 56% retained normal VEMP test results, thus it can be concluded that the impact of CI surgery on the vestibular apparatus was not clinically significant. It is worth noting that some conditions such as the use of ototoxic drugs or Meniere’s disease might be present in CI users, and could limit the interpretation of abnormal balance tests in case testing was done only postoperatively. However, the studies did not report detailed patients’ medical history to be conclusive.

It is important to note that some studies were performed by the same group (Nordfalk et al. [19, 21], Krause et al. [22–24], and Kluenter et al. [6, 25]). The authors were contacted to verify whether these studies have an overlap. Nordfalk et al. have different sets of patient populations, so they do not overlap. Kluenter et al. had 12 patients who participated in both studies. No response was received from Krause et al.

We found that CI surgery can significantly affect the results of both the caloric test and VEMP test. This finding is in accord with the systematic review of Kuang et al. [26], where they found that 37% of patients had reduced reflex, and 34% had caloric asymmetry after CI surgery. Other authors [27, 28] reported that one-third of CI recipients complain of dizziness after surgery. A recent review aimed at determining the best test to evaluate vestibular function before and after CI surgery was published by Abouzayd et al., [29]. They found that the caloric test was least sensitive, VEMP results were most often impaired, and HIT results were generally conserved. Our study provides a quantified evidence that CI surgery can significantly affect some vestibular test results (although it might not be clinically significant, as evident from the pre- and postoperative DHI scores). It also provides estimates of vestibular dysfunction in CI candidates. The current study confirms that it is important to pursue a case-by-case approach with CI surgery candidates, based on each patient’s history and symptoms.

To summarize, several factors can contribute to the variability of the results within and between the vestibular function tests, both before and after CI surgery, that are difficult to control for. Those factors include age and etiology of hearing loss, the surgical technique used, and the incidence of trauma to the inner ear. Because congenital, genetic, and post-meningitis hearing loss is more common in children, a separate analysis of pediatric vestibular function before and after CI surgery, and comparing the results to adults, would be a useful area of future research.

## Conclusion

According to the results of the current meta-analysis, CI surgery can significantly affect the results of caloric as well as VEMP tests. No significant effect was detected in HIT results, posturography, or DHI scores. Drawing a definitive conclusion is rather difficult for a number of reasons, such as heterogeneity in study design, variability among patient populations, pre-existing condition, and measurement and reporting differences. Whilst studies showed that some postoperative scores were worse after CI, the proportion of patients affected appears low. Age and etiology of hearing loss appear to affect the vestibular function after CI surgery. Nonetheless, the possible effects of CI surgery on the vestibular system should be communicated to CI recipients before surgery.

## Appendix

**Table 2:** Number of subjects with normal and abnormal testing results before and after surgery in studies included in the meta-analysis for the HIT test

Study	Year	Normal pre	Abnormal pre	Normal post	Abnormal post	Number pre	Number post
Basta [12]	2008	18	0	18	0	18	18
Batuecas [8]	2015	30	0	20	10	30	30
Jutila [32]	2012	19	25	15	29	44	44
Melvin [5]	2009	14	0	10	0	14	10
Migliaccio [10]	2005	14	2	10	1	16	11
Vankatova [9]	2014	50	0	43	7	50	50

Normal pre = number of individuals with normal test results before surgery. Abnormal Pre = number of individuals with abnormal test results before surgery. Normal post = number of individuals with normal test results after surgery. Abnormal post = number of individuals with abnormal test results after surgery. Number pre = number of individuals tested before surgery. Number post = number of individuals tested after surgery

**Table 3:** Number of subjects with normal and abnormal testing results before and after surgery in studies included in the meta-analysis for the caloric test

Study	Year	Normal Pre	Abnormal pre	Normal post	Abnormal post	Number pre	Number post
Abramides [18]	2014	14	34	8	40	24	24
Basta [12]	2008	16	2	15	3	18	18
Batuecas [8]	2015	30	0	27	3	30	30
Bonucci [15]	2008	15	23	9	29	38	38
Brey [14]	1995	8	9	5	12	17	17
Ito [31]	1998	18	37	11	44	55	55
Katsiari [2]	2013	7	13	4	16	20	20
Kiyomizu [33]	2000	13	10	7	16	23	23
Kluenter [6]	2009	18	6	21	3	24	24
Kluenter [25]	2010	41	11	44	8	52	52
Krausea [22]	2009	25	20	15	27	45	42
Krauseb [23]	2009	35	21	13	40	56	53
Krause [24]	2010	13	9	8	14	32	32
Louza [34]	2014	30	11	8	33	41	41
Melvin [5]	2009	14	6	15	1	20	16
Nordfalk [19]	2015	20	10	13	17	30	30
Rossi [35]	1998	8	24	7	25	32	32
Todt [36]	2008	48	14	36	26	62	62
Wagner [17]	2010	17	5	16	6	22	22

Normal pre = number of individuals with normal test results before surgery. Abnormal Pre = number of individuals with abnormal test results before surgery. Normal post = number of individuals with normal test results after surgery. Abnormal post = number of individuals with abnormal test results after surgery. Number pre = number of individuals tested before surgery. Number post = number of individuals tested after surgery

**Table 4:** Number of subjects with normal and abnormal testing results before and after surgery in studies included in the meta-analysis for the VEMP test

Study	Years	Normal. pre	Abnormal. pre	Normal. post	Abnormal. post	Number. pre	Number. post
Basta [12]	2008	16	2	6	12	18	18
Coordes [13]	2012	17	0	14	3	17	17
Ernst [30]	2006	12	6	4	14	18	18
Katsiari [2]	2013	10	10	4	16	20	20
Krause [24]	2010	14	16	8	22	30	30
Louza [34]	2014	29	12	11	30	41	41
Melvin [5]	2009	12	7	11	5	19	16
Nordfalk [21]	2014	9	3	5	7	12	12
Nordfalk [19]	2015	25	8	13	20	33	33
Robard [11]	2015	22	12	9	25	34	34
Todt [36]	2008	39	23	28	34	62	62
Wagner [17]	2010	22	18	17	23	20	20

Normal pre = number of individuals with normal test results before surgery. Abnormal Pre = number of individuals with abnormal test results before surgery. Normal post = number of individuals with normal test results after surgery. Abnormal post = number of individuals with abnormal test results after surgery. Number pre = number of individuals tested before surgery. Number post = number of individuals tested after surgery

**Table 5:** Test results (Sensory Organization test scores) before and after surgery in studies included in the meta-analysis for the posturography test

Study	Year	Mean1	SD1	Number1	Mean2	SD2	Number2
Brey [14]	1995	46.99	25.68	22	45	31.04	22
Brey [14]	1995	43.5	22.1	22	42.17	28.76	22
Buchman [3]	2004	39	26	82	40	27	67
Buchman [3]	2004	33	26	82	31	26	67

Mean1 = mean scores before surgery. SD1 = scores standard deviations before surgery. Number1 = number of patients tested before surgery. Mean2 = mean scores after surgery. SD2 = scores standard deviations after surgery. Number2 = number of patients tested after surgery

**Table 6:** Test results (DHI scores) before and after surgery in studies included in the meta-analysis for the DHI test

Study	Year	Mean1	SD1	Number1	Mean2	SD2	Number2
Basta [12]	2008	5	1.41	18	64	14.14	18
Buchman [3]	2004	5	8	78	4	8	66
Migliaccio [10]	2005	10.54	11.76	11	9.09	11.18	11
Wagner [17]	2010	14.9	24.4	20	17.6	22.2	20

Mean1 = mean scores before surgery. SD1 = scores standard deviations before surgery. Number1 = number of patients tested before surgery. Mean2 = mean scores after surgery. SD2 = scores standard deviations after surgery. Number2 = number of patients tested after surgery

## Abbreviations

CI: Cochlear Implant
DHI: Dizziness Handicap Inventory
HIT: Head Impulse Test
MD: Mean Difference
RR: Relative Risk
SOT: Sensory Organization Test
VEMP: Vestibular Evoked Myogenic Potential

## Footnotes

1 Todt et al., 2008 [36] claimed that the use of round window approach for electrode insertion would decrease the probability of loss of vestibular function postoperatively, compared to the standard cochleostomy approach.
